# Harmine enhances the activity of the HIV-1 latency-reversing agents ingenol A and SAHA

**DOI:** 10.1242/bio.052969

**Published:** 2020-12-22

**Authors:** Jared P. Taylor, Lucas H. Armitage, Daniel L. Aldridge, Melanie N. Cash, Mark A. Wallet

**Affiliations:** Department of Pathology, Immunology & Laboratory Medicine, University of Florida, Gainesville, FL 32610, USA

**Keywords:** Harmine, Latency, HIV

## Abstract

Infection with human immunodeficiency virus 1 (HIV-1) remains incurable because long-lived, latently-infected cells persist during prolonged antiretroviral therapy. Attempts to pharmacologically reactivate and purge the latent reservoir with latency reactivating agents (LRAs) such as protein kinase C (PKC) agonists (e.g. ingenol A) or histone deacetylase (HDAC) inhibitors (e.g. SAHA) have shown promising but incomplete efficacy. Using the J-Lat T cell model of HIV latency, we found that the plant-derived compound harmine enhanced the efficacy of existing PKC agonist LRAs in reactivating latently-infected cells. Treatment with harmine increased not only the number of reactivated cells but also increased HIV transcription and protein expression on a per-cell basis. Importantly, we observed a synergistic effect when harmine was used in combination with ingenol A and the HDAC inhibitor SAHA. An investigation into the mechanism revealed that harmine, when used with LRAs, increased the activity of NFκB, MAPK p38, and ERK1/2. Harmine treatment also resulted in reduced expression of HEXIM1, a negative regulator of transcriptional elongation. Thus, harmine enhanced the effects of LRAs by increasing the availability of transcription factors needed for HIV reactivation and promoting transcriptional elongation. Combination therapies with harmine and LRAs could benefit patients by achieving deeper reactivation of the latent pool of HIV provirus.

## Abstract (precis)

**Summary:** The plant-derived compound harmine shows efficacy in enhancing the effects of existing HIV latency-reversing drugs.

## INTRODUCTION

Infection with human immunodeficiency virus 1 (HIV-1) remains incurable because of the ability of this virus to integrate its genetic material permanently into the host genome of infected cells. The single-stranded RNA genome of HIV requires a stable intermediate in the form of an integrated proviral DNA to complete its life cycle. This integrated DNA is subject to the same regulatory mechanisms as host genes including requirements for active transcription factors as well as epigenetic control of chromatin structure and accessibility (Siliciano and Greene, 2011).

Like host genes, the HIV genome may enter a state of non-expression wherein viral mRNA and proteins are not expressed (Folks et al., 1986). Whether this non-expression is through neglect (lack of stimuli required to drive the HIV long terminal repeat, LTR, promoter) or active suppression (binding of inhibitory proteins to the LTR or epigenetic silencing of the locus), the outcome is the same – HIV is not produced, and the virus remains hidden from host immunity. In this state, HIV endures for as long as the infected cell and all progeny from future cell divisions. If replication-competent proviral genomes are harbored, an individual is at risk for viral reactivation.

Even after HIV replication has been pharmacologically suppressed so that the virus is undetectable in peripheral blood, cessation of treatment leads to the resumption of the HIV/AIDS clinical progression (Davey et al., 1999). Elimination of the latent reservoir or significant reduction of the size of the reservoir are seen as the only real hopes for a sterilization or functional cure, respectively. A large body of work is focused on pharmacological strategies to ‘purge’ the latent reservoir. The most widely studied approach is known as ‘shock and kill’ or ‘kick and kill’ (Deeks, 2012; Hamer, 2004). The goal is to simultaneously reactivate all (or most) replication-competent latent HIV while maintaining antiretroviral therapy (ART). Ideally, upon re-expression of viral mRNA and proteins, the reservoir cells will die through either cytopathic effects of the virus or through immune mechanisms, such as cytotoxic T lymphocytes (CTL) or natural killer (NK) cells. What has been lacking is a safe and effective pharmacological method to potently reactivate latent HIV *in vivo.*

HIV latency is predominantly controlled by two basic mechanisms: chromatin accessibility and transcription factor expression/activation/localization. Epigenetic regulation of the HIV integration site through modification of histone proteins by histone deacetylase (HDAC) enzymes effectively silences HIV mRNA expression. HDAC inhibitors such as vorinostat (SAHA) can elicit HIV replication from latently infected cells *in vitro* (Archin et al., 2009a,b; Contreras et al., 2009; Lehrman et al., 2005)*. In vivo*, vorinostat can induce some reactivation and increase plasma HIV RNA in subjects receiving ART (Archin et al., 2012). However, thus far HDAC inhibitors have not been able to significantly reduce the pool of latently infected cells.

Transcription of HIV mRNA relies on the interaction of the viral protein Tat and the pTEFb complex. The pTEFb complex is a positive regulator of transcription elongation and the interaction of Tat with pTEFb is required for efficient elongation of HIV transcripts (Mancebo et al., 1997; Zhu et al., 1997). The activation of host transcription factors such as NFκB and MAPK are also required for reactivation of HIV from latency. These transcription factors are normally sequestered in the cytoplasm as inactive proteins. Numerous early studies of HIV latency have focused on treatments that stimulate T cell activation including cytokines (IL-2 and IL-7), ligation of surface proteins (PHA, anti-CD3) or chemical stimulators of signaling pathways such as protein kinase C (PKC) agonists. NFκB, in particular, is a potent inducer of HIV mRNA expression and several PKC agonists have been validated for their ability to elicit reactivation of latent HIV. These treatments are even more potent when paired with complementary drugs that target HDACs or BRD4. Other transcription factors such as SP1, STATs, and IRFs play roles in regulating the HIV LTR. The NFAT family of transcription factors may also regulate HIV latency since the HIV LTR contains NFAT transcription factor binding sites, and NFAT can enhance HIV mRNA expression. NFAT, like NFκB, is sequestered as an inactive protein in the cytoplasm unless activated by specific upstream signals. However, unlike NFκB, NFAT is hyperphosphorylated in its inactive state (Macian, 2005). This hyperphosphorylation is driven by a dual-specificity kinase DYRK1A (Arron et al., 2006; Gwack et al., 2006) and NFAT only becomes de-phosphorylated following calcium-dependent calmodulin signaling (Macian, 2005).

Harmine is a naturally-occurring tricyclic β-carboline alkaloid with hallucinogenic properties that is derived from the plant *Banisteriopsis caapi* as well as others (Patel et al., 2012). Harmine has been shown to be an inhibitor of DYRK1A (Adayev et al., 2011; Bain et al., 2007; Göckler et al., 2009). Harmine's primary target is monoamine oxidase A (MAO-A) (Blum et al., 1964; Slotkin and DiStefano, 1970; TUNG et al., 1965; Yasuhara, 1974), but a well-reported role for inhibition of DYRK1A leading to enhanced NFAT activity has been reported (Adayev et al., 2011; Bain et al., 2007; Egusa et al., 2011; Göckler et al., 2009). The crystal structure of harmine complexed with DYRK1A has been solved, confirming its binding to the ATP-binding pocket of DYRK1A (Ogawa et al., 2010). Because of its ability to augment NFAT signaling, we undertook a study of harmine and other DYRK1A inhibitors to determine whether these compounds could enhance HIV reactivation alone or in combination with other known latency-reversing agents (LRAs). We hypothesized that DYRK1A inhibitors would augment reactivation with LRAs through increased NFAT availability. Here, we report that harmine and another DYRK1A inhibitor, INDY (Ogawa et al., 2010), boost HIV reactivation by PKC agonists. Interestingly, the effect was independent of NFAT activity; harmine was effective at boosting LRAs even when no evidence of NFAT activity was detected. In addition, CRISPR knockout of DYRK1A did not mimic the effects of harmine or modulate the efficacy of harmine for boosting HIV reactivation, Instead, we found that harmine enhances MAPK and NFκB signalling, leading to increased HIV transcription. Using whole-genome microarray we observed that harmine modulates expression of key pTEFb components. *HEXIM1* expression is significantly downregulated by harmine whereas the cyclin *CCNT2* was upregulated. We conclude that harmine regulates pTEFb complex heterogeneity and establishes an environment that is more conducive to HIV reactivation. Combination treatments with harmine and LRAs may prove efficacious *in vivo*.

## RESULTS

### DYRK1A inhibitors enhance the efficacy of T-cell activating PKC agonists

**Fig. 1.**
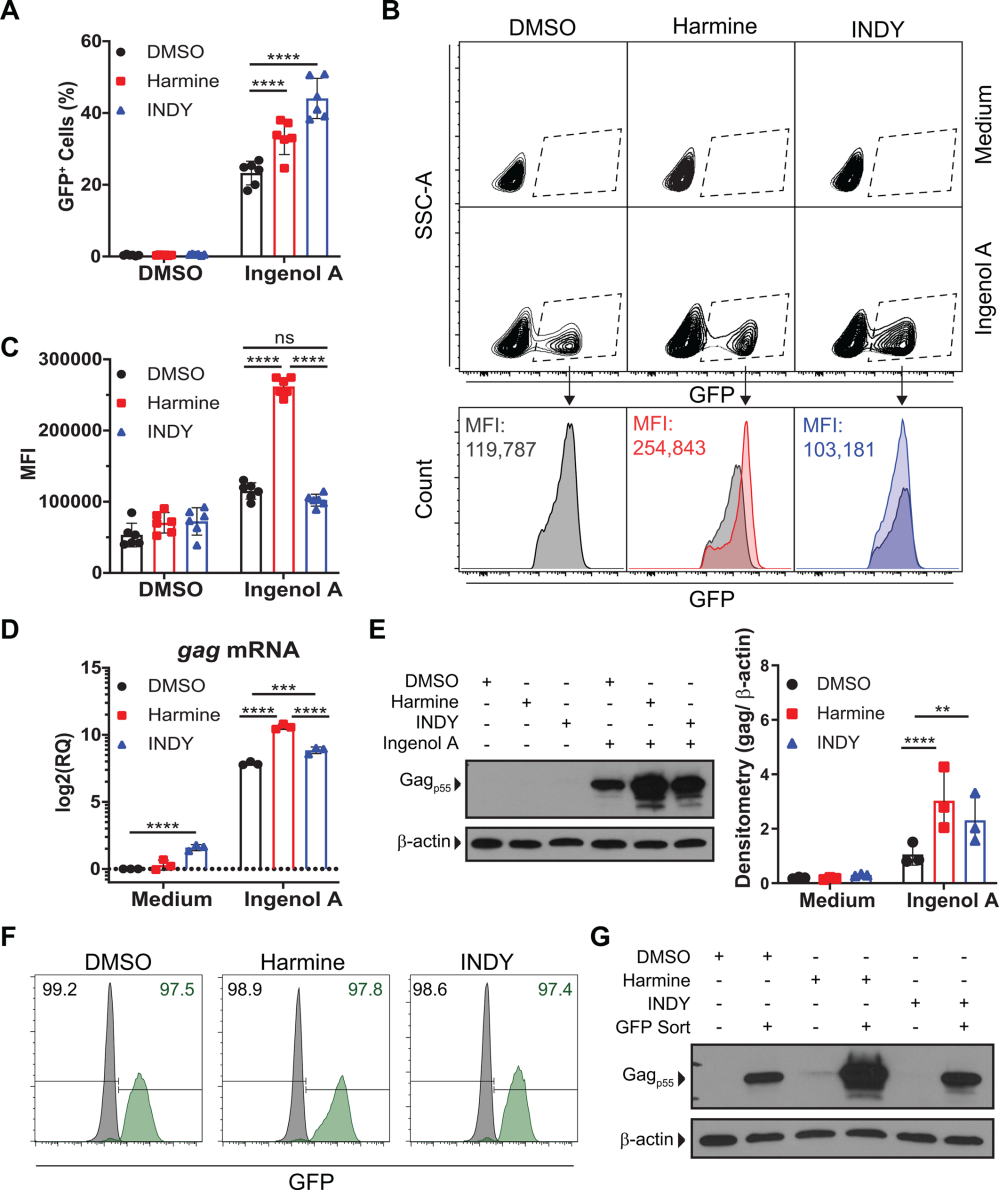
**DYRK1A inhibitors enhance the efficacy of PKC agonist ingenol A.** (A) The percentage of GFP^+^ J-Lat 5A8 cells after reactivation with ingenol A in the presence of inhibitors (*n*=6), (B) representative flow cytometry plots of the mean fluorescence intensity of GFP^+^ cells, and (C) summary mean fluorescence intensity data (*n*=6). (D) *gag* mRNA expression determined by RT-qPCR. (E) Gag protein expression determined by western blot with representative blot and densitometry (*n*=3). GFP^+^ and GFP^−^ cells were sorted after reactivation with ingenol A. (F) Post-sort purity measured by flow cytometry. The percentage of GFP^−^ population (black) and the GFP^+^ population (green). (G) Gag protein expression of post-sort GFP^+^ cells measured by western blot. Error bars represent standard deviation. Statistical analysis was performed by two-way ANOVA and corrected for multiple comparisons by Tukey's test. **P<0.01; ***P<0.001; ****P<0.0001; ns, not significant.

PKC agonists have been previously shown to reactivate latent HIV (Brogdon et al., 2016; Díaz et al., 2015; Jiang et al., 2015; Laird et al., 2015; Martínez-Bonet et al., 2015; Perez et al., 2010; Reuse et al., 2009; Williams et al., 2004). We expected reactivation with PKC agonists to be enhanced by combinatorial treatment with DYRK1A inhibitors, harmine or INDY (Fig. S1A). To test this, we used J-Lat 5A8 reporter cells, which are latently infected with a full-length provirus that expresses GFP in place of Nef as an indicator of LTR activity. J-Lat 5A8 cells were pretreated with harmine or INDY and reactivated with the PKC agonist ingenol A. Both harmine and INDY failed to cause any measurable HIV reactivation when used alone, however, harmine and INDY boosted the reactivation effects of ingenol A (Fig. 1A). This effect could be seen with doses as low as 5 µM in J-Lat cells and as low as 2.5 µM in primary CD4+ T cells (Fig. S1B,C). Toxicity caused by harmine and INDY was seen with doses above 25 µM (Fig. S1D), therefore, a dose of 20 µM was used for experiments.

When analyzing flow cytometry data, we recognized a unique phenomenon in the J-Lat reactivation experiments. Not only was harmine increasing the frequency of cells that became GFP^+^, but it also appeared that the brightness of each GFP^+^ cell was increased when harmine was included (Fig. 1B,C). J-Lat cells were then activated with increasing doses of ingenol A, PMA, or TNF in the presence of DMSO, harmine, or INDY. Both harmine and INDY boosted the frequency of ingenol A or PMA-activated cells whereas only INDY had a positive effect on reactivation with TNF treatment (Fig. S2). When gating on only the GFP^+^ cells, it became clear that harmine increases the amount of GFP expressed in each reactivated cell (Fig. S2). This finding suggests that harmine works through a mechanism that is unique from INDY. It also seems to indicate that the boosting effect of harmine is working through at least two biological pathways, one that increases the sensitivity of cells to activating stimuli (reduced dose of stimulus required to activate HIV) and a second pathway that enhances the magnitude of HIV LTR activity in cells where reactivation occurs.

For a more direct measure of HIV-encoded genes/proteins, J-Lat cells were activated with ingenol A in the presence or absence of harmine, or INDY. Here, like GFP, *gag* mRNA expression was induced by ingenol A+DMSO and the expression was markedly increased with the addition of harmine (Fig. 1D). Harmine alone failed to induce *gag* expression. However, INDY alone did result in increased gag expression (Fig. 1D) even though INDY treatment alone did not result in increased activation of J-Lat 5A8 cells (Fig. 1A). Next, western blot analysis was performed to measure gag protein expression. Gag protein expression was induced by ingenol A alone and markedly increased with the addition of harmine or INDY. The effect was stronger for harmine than with INDY (Fig. 1E). To better measure the amount of gag being expressed on a per cell basis, flow cytometry sorting was used to separate GFP^+^ versus GFP^−^ cells after ingenol A treatment in the presence or absence of harmine or INDY. Gag protein expression was measured in the GFP^+^ and GFP^−^ cells by western blot. The GFP^+^ cells that were treated with ingenol A+harmine expressed more gag protein than GFP^+^ cells that were treated with ingenol A+INDY (Fig. 1F&G). Again, these findings indicate that harmine is mediating two distinct effects on HIV latency. One effect increases the sensitivity of cells to activating LRAs whereas a second effect increases the expression level of HIV genes/proteins. This finding leads to two key questions: does harmine enhance HIV reactivation through enhanced NFAT activity? And is DYRK1A the target of harmine in HIV latency models?

### Harmine boosts HIV reactivation independent of NFAT

DYRK1A negatively regulates NFAT by hyperphosphorylation, which excludes NFAT from the nucleus. Only through calcium-dependent calmodulin activation is NFAT de-phosphorylated resulting in nuclear translocation and activation of NFAT dependent genes such as IL-2. The HIV LTR contains at least two NFAT binding motifs and since harmine is a known inhibitor of DYRK1A, we wanted to determine if the anti-latency effects of harmine are partly due to enhanced NFAT activity.

**Fig. 2.**
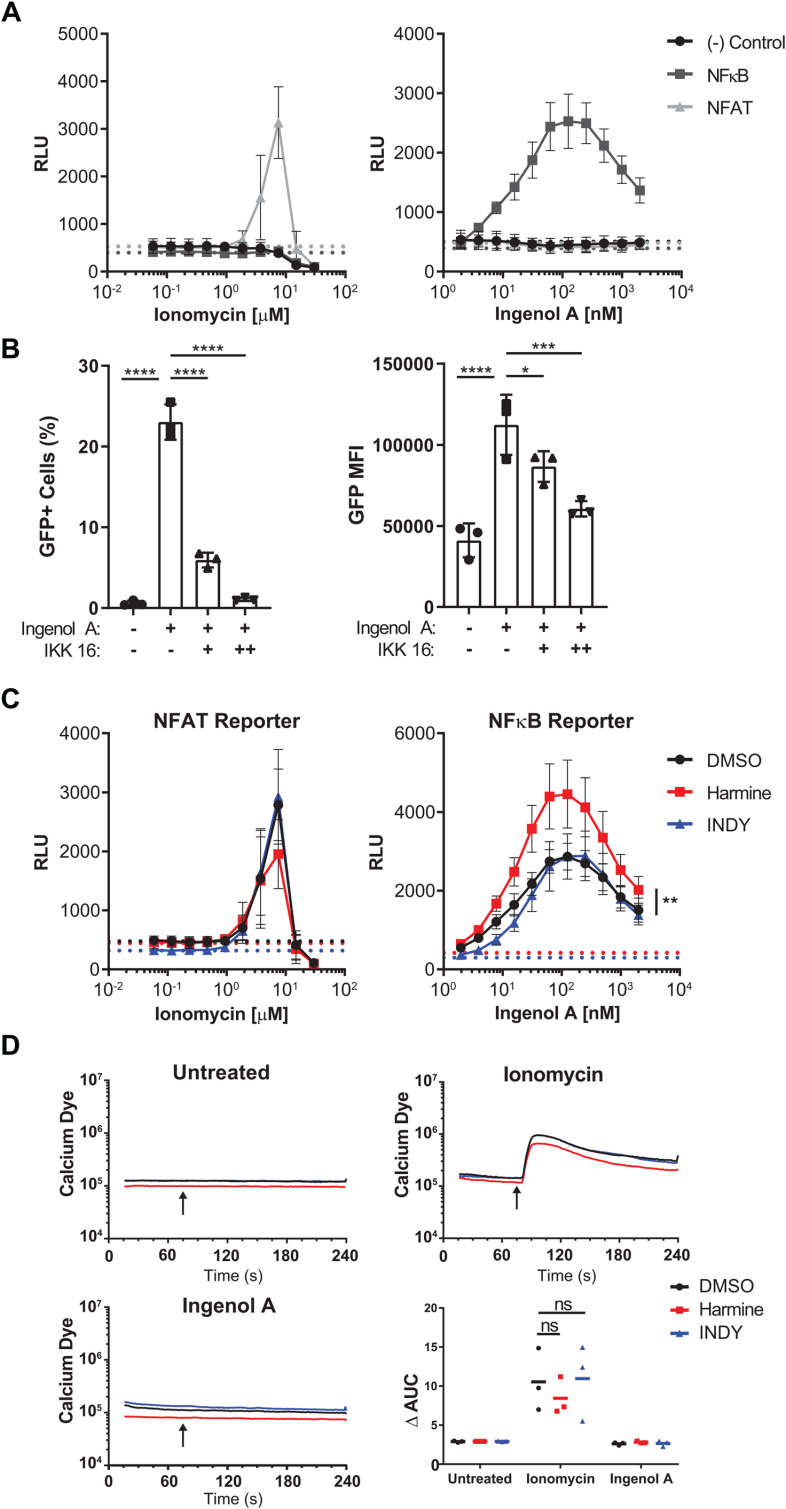
**Harmine's boosting effect is through NFκB, not NFAT.** (A) Ionomycin or ingenol A were titrated on J-Lat 5A8 luciferase reporter cells. The dashed lines indicate the luciferase activity with no ionomycin or ingenol A treatment. (B) NFAT or NFκB luciferase reporter cells reactivated by ionomycin or ingenol A in the presence of inhibitors. Dashed lines represent luciferase activity with no ionomycin or ingenol A treatment. (C) J-Lat 5A8 cells were stained with Calcium Sensor Dye eFluor 514 (2 µM) and treated with DMSO, harmine, or INDY for 30 min. Geometric mean fluorescence was measured by flow cytometry for 240 s. Ionomycin (250 nM) or ingenol A (50 nM) was added after 75 s (arrows). Plots show mean geometric fluorescence intensity over time and the change in area under the curve (*n*=3). (D) J-Lat 5A8 cells were pretreated with DMSO or an IκB kinase inhibitor, IKK 16, at 1 µM (+) or 10 µM (++) followed by ingenol A treatment (31.25 nM) for 18 h (*n*=3). Error bars represent standard deviation. Statistical analysis was performed by one-way-ANOVA corrected for multiple comparisons with Dunnett's test (D) and one-way-ANOVA of the area under the curve corrected for multiple comparisons by Tukey's test (C). *P<0.05; ***P<0.001; ****P<0.0001; ns, not significant; RLU, relative light units.

First, we wanted to determine whether harmine affects NFAT activity in T cells. Jurkat T cells were transduced with lentiviral luciferase reporter constructs for NFAT, NFκB or a negative control virus with luciferase gene but no promoter. When reporter cells were treated with ionomycin alone, a well-known activator of calmodulin and NFAT, only the NFAT reporter cells expressed luciferase (Fig. 2A). When reporter cells were treated with ingenol A alone, only the NFκB cells expressed luciferase (Fig. 2A). This suggests that ingenol A-mediated reactivation of J-Lat 5A8 cells is dependent on NFκB, not NFAT. Furthermore, treatment of J-Lat 5A8 cells with IKK16, an inhibitor of NFκB signaling, significantly reduced the percentage of reactivated cells as well as the MFI of GFP^+^ cells in a dose-dependent manner (Fig. 2B). To determine if harmine was influencing NFAT or NFκB activity, NFAT and NFκB reporter cells were treated with ingenol A with harmine or INDY. Harmine had no effect on ionomycin-induced luciferase in the NFAT reporter cells. However, harmine significantly boosted luciferase activity in the NFκB reporter cells compared to INDY. INDY had no effect on luciferase expression in any of the cell lines (Fig. 2C). These data suggest that the boosting effects of harmine and INDY are through different mechanisms.

Second, we wanted to determine whether harmine or INDY affected calcium flux upstream of NFAT activation. Jurkat cells were stained with a calcium-sensitive dye and treated with ionomycin or ingenol A. Ionomycin induced a calcium flux as expected but ingenol A did not. Neither harmine nor INDY had any effect on calcium flux regardless of agonist treatment (Fig. 2D). Harmine is primarily affecting the NFκB pathway with no significant involvement of NFAT or the calcium flux. Thus, harmine and INDY are working through different mechanisms. Harmine appears to be acting upon the NFκB pathway.

**Fig. 3.**
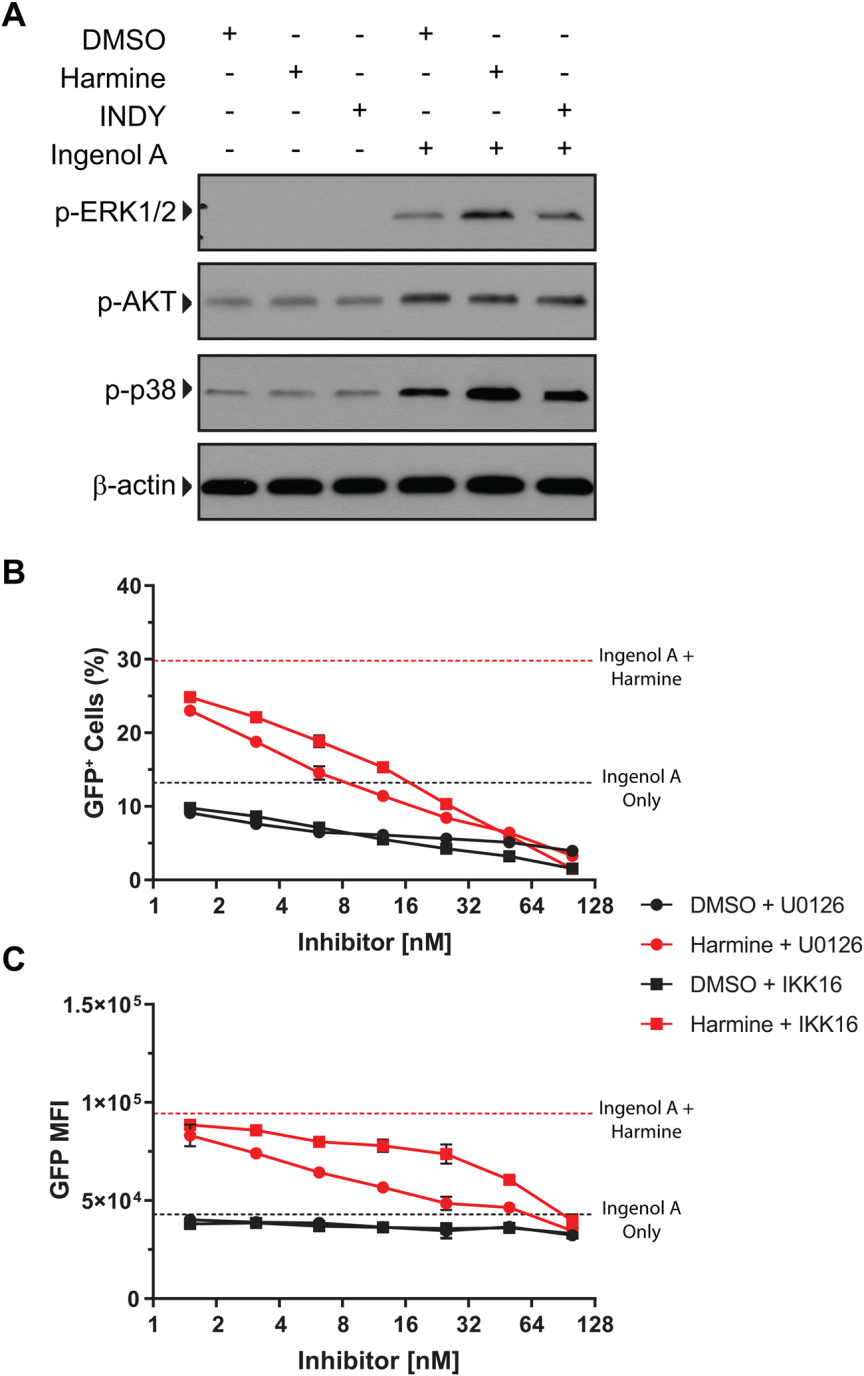
**Harmine boosts phospho-ERK1/2 and phospho-p38 levels after ingenol A stimulation.** J-Lat 5A8 cells were reactivated with ingenol A (31.25 nM) in the presence of inhibitors. (A) Whole cell lysates were analyzed by western blot for phospho-ERK1/2, phospho-AKT, and phospho-p38. (B) J-Lat 5A8 cells were pretreated with DMSO, a MEK inhibitor (U0126), or an IκB kinase inhibitor (IKK 16) for 30 min followed by overnight treatment with ingenol A (100 nM). GFP expression was assessed by flow cytometry. The percentage and (C) mean fluorescence intensity of the GFP^+^ cells are shown. Dashed lines represent treatment with ingenol A alone.

We next wanted to determine whether harmine was only affecting the NFκB pathway or if it was also affecting other pathways downstream of PKC. J-Lat cells cultured with PMA +/− harmine were analyzed for phospho-ERK1/2, phospho-MAPKp38, and phospho-AKT levels. We found that harmine markedly boosted phospho-ERK1/2 and phospho-MAPKp38 levels induced by PMA (Fig. 3A). We found no difference in levels of phospho-AKT (Fig. 3A). Inclusion of an ERK inhibitor, U0126, resulted in dose-dependent suppression of ingenol A-induced HIV reactivation (Fig. 3B). Importantly, while the inhibitor markedly reduced the frequency of GFP^+^ cells, it also reduced the MFI of GFP^+^ cells (Fig. 3C). This reduction in the MFI of the GFP^+^ cells was more pronounced than the reduction in MFI seen with the inhibition of NFκB. Thus, it appears that harmine boosts the frequency of GFP^+^ cells by enhancing sensitivity to PKC agonists as well as increases the magnitude of LTR activity independent of a strong activating signal.

### Harmine boosts HIV reactivation in the absence of DYRK1A

**Fig. 4.**
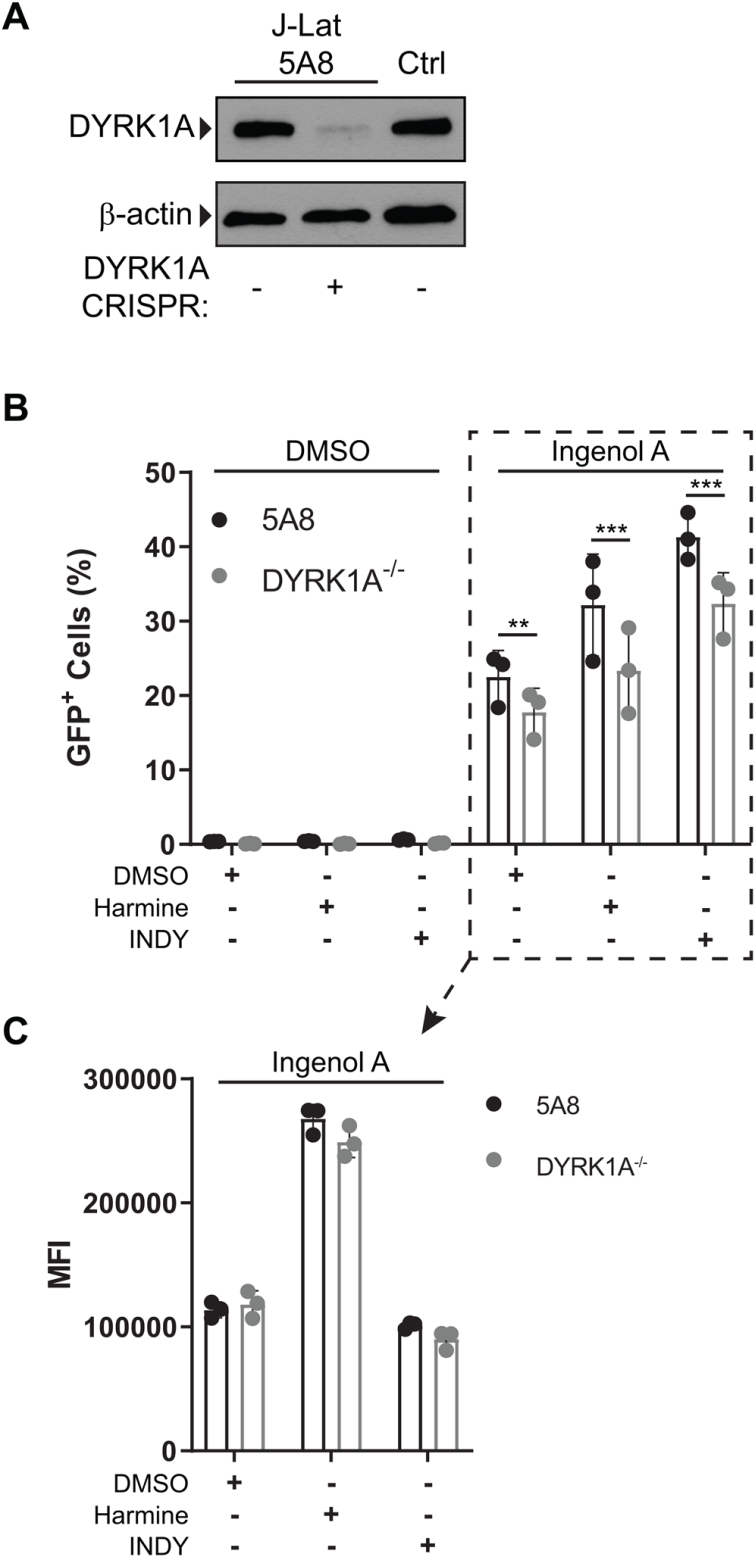
**Harmine boosts independently of DYRK1A.** DYRK1A was knocked out with CRISPR/Cas9 in J-Lat 5A8 cells. (A) Western blot of J-Lat 5A8 cells after treatment with CRISPR compared to Jurkat cells (Ctrl). The CRISPR knockout cells were reactivated with ingenol A (31.25 nM) in the presence of inhibitors. (B) The percentage of GFP^+^ cells and the (C) mean fluorescence intensity of GFP^+^ cells was measured by flow cytometry. Error bars represent standard deviation. Statistical analysis was performed by two-way ANOVA corrected for multiple comparisons with Bonferroni's test. **p<0.01; ***p<0.001.

Since we observed that the boosting effect of harmine was independent of NFAT activity and was associated with altered MAPK signaling, we wanted to confirm that the increase in GFP^+^ cells and the increase in the MFI of GFP^+^ cells by harmine was DYRK1A-dependent. To test this, a J-Lat cell line lacking DYRK1A expression was derived by CRISPR/Cas9 gene targeting (Fig. 4A). If the phenotype caused by harmine were due to its interactions with DYRK1A, then we anticipated that J-Lat cells lacking DYRK1A would behave similarly to harmine treated cells with increased GFP expression in response to activating LRAs. DYRK1A knockout did lead to a significant decrease in the percentage of GFP^+^ cells. However, this decrease was also seen in knockout cells not treated with an inhibitor (DMSO control) (Fig. 4B). Interestingly, DYRK1A knockout had no effect on the MFI of GFP^+^ cells (Fig. 4C). Although DYRK1A knockout led to a decrease in the number of GFP^+^ cells, treatment with harmine and INDY still resulted in an increase in GFP^+^ cells (Fig. 4B). This suggests that while DYRK1A expression may be involved in reactivation of cells (Fig. 4B) it is not required and is not the pathway targeted by harmine and INDY that results in increased HIV reactivation.

### Harmine downregulates HEXIM1 expression

**Fig. 5.**
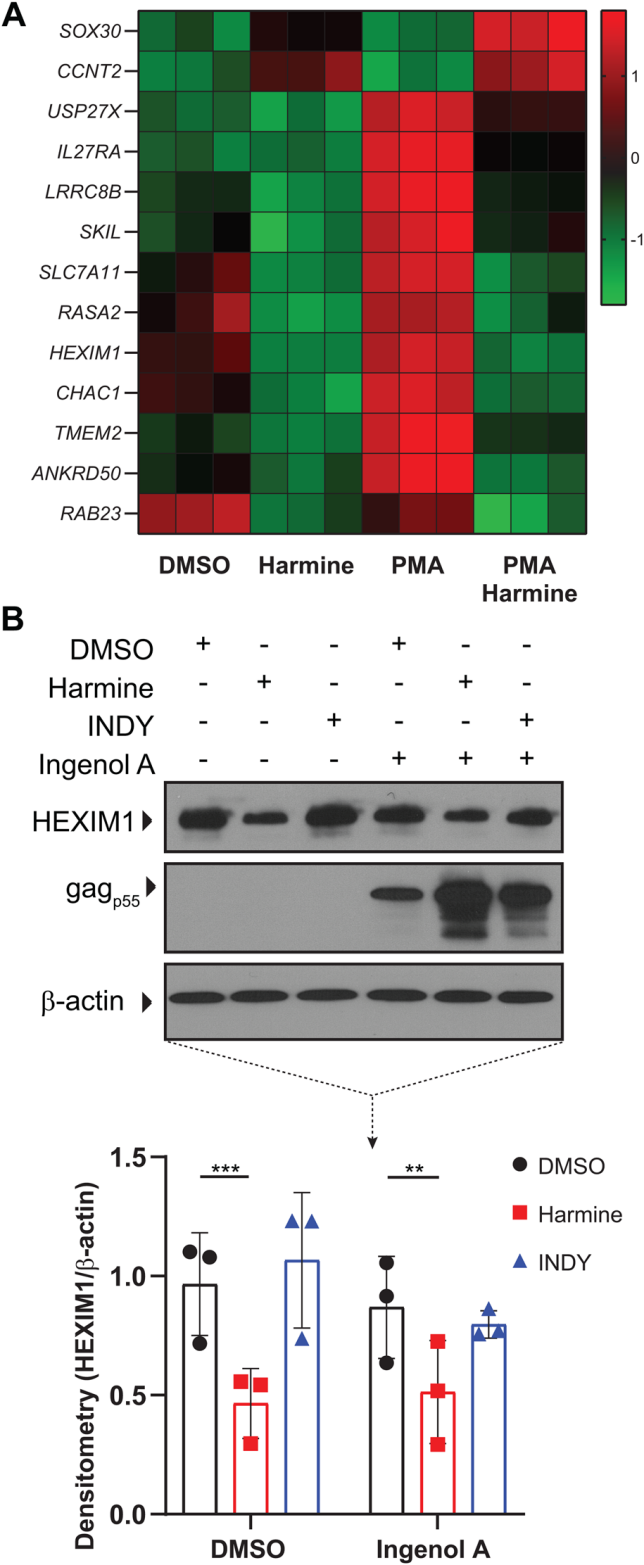
**Harmine downregulates HEXIM1 expression.** J-Lat 5A8 cells were treated pretreated with DMSO, harmine, or INDY for 30 min followed by PMA stimulation for 2 hours and analyzed by whole transcriptome microarray. (A) Hierarchical clustering of transcripts that were upregulated or downregulated at least twofold between PMA and harmine+PMA treatments. (B) Representative western blot analysis of HEXIM1 expression and densitometry (*n*=3). Error bars represent standard deviation. Statistical analysis was performed by two-way ANOVA corrected for multiple comparisons with Tukey's test. **P<0.01; ***P<0.001.

To better understand how harmine is modulating HIV reactivation, we used whole-genome microarray to determine which genes were differentially expressed in cells treated with harmine. J-Lat cells were treated with DMSO, harmine, PMA, or harmine+PMA. Microarray analysis showed that there were 35 transcripts that were significantly upregulated or downregulated by at least twofold between PMA and PMA+harmine treatments. A complete list of the 35 significantly upregulated or downregulated genes are presented in Table S1. Fourteen of these transcripts correspond to coding genes (Fig. 5A and Table 1) and the remaining 21 transcripts were non-coding transcripts. The gene that was upregulated the most in PMA+harmine treatment compared to PMA treatment alone was *CCNT2*, which codes for the cyclin T2 protein. The gene that was downregulated the most in PMA+harmine treatment compared to PMA treatment alone was *HEXIM1.* Both genes code for proteins that play a role in transcriptional elongation (Chen et al., 2018). The HIV-encoded transcription factor Tat competes with HEXIM1 for binding to the pTEFb complex to promote HIV transcription (Barboric et al., 2007). Downregulation of *HEXIM1* would thus result in less negative regulation of the pTEFb complex resulting in more Tat binding to pTEFb promoting transcript elongation. Similarly, upregulation of cyclin T2 would be expected to increase elongation of transcripts. This combination of effects on transcription explains why harmine treatment not only increases the percentage of reactivated cells but also increases the number of viral transcripts on a per-cell basis.

**Table 1.**
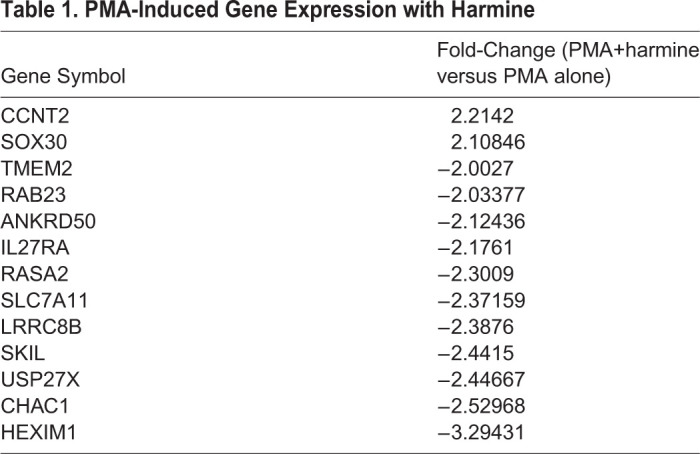
**PMA-Induced Gene Expression with Harmine**

To confirm the microarray findings, we treated J-Lat cells with ingenol A in the presence or absence of harmine or INDY. Western blot analysis showed that harmine treatment alone or with ingenol A leads to a significant reduction in HEXIM1 protein expression, whereas INDY treatment has no effect (Fig. 5B). The decrease in HEXIM1 protein expression corresponded to an increase in the expression of gag protein. Thus, harmine treatment alone downregulates HEXIM1 and boosts the efficacy of subsequent LRA stimulation.

### Harmine boosts the efficacy of SAHA-induced HIV reactivation

**Fig. 6.**
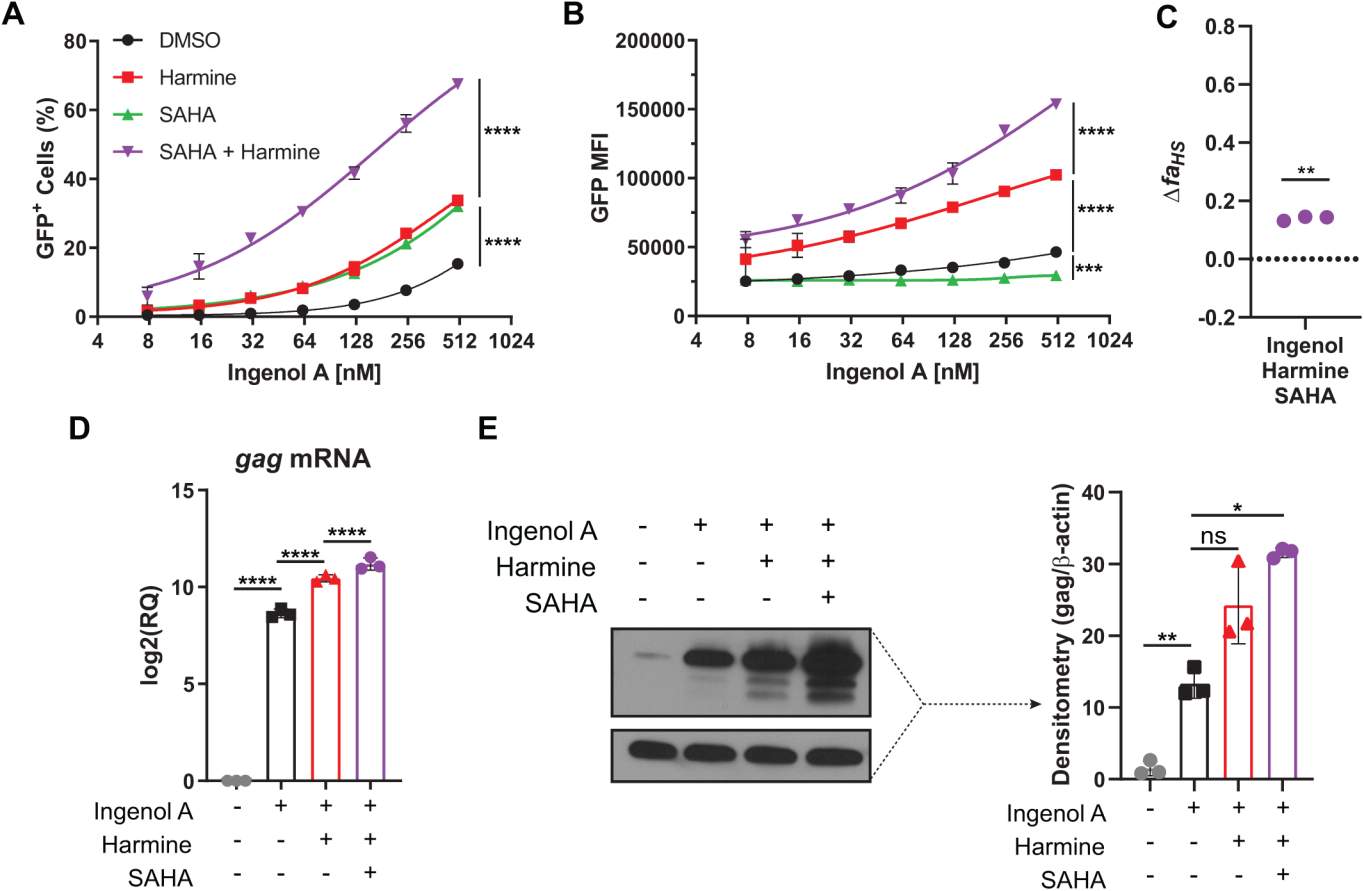
**SAHA and harmine act synergistically to boost ingenol A activation of J-Lat cells.** J-Lat 5A8 cells were pretreated with DMSO, harmine, SAHA, or SAHA+harmine for 30 min followed by ingenol A (31.25 nM) stimulation for 18 h. (A) The percentage of GFP^+^ cells and (B) the mean fluorescence intensity (*n*=3). (C) Synergy calculated using the Bliss Independnce Model. Expression of *gag* mRNA and gag protein were measured by (D) qPCR and (E) western blot, respectively (*n*=3). Statistical analysis was performed by calculating the area under the curve and performing a two-way ANOVA corrected for multiple comparisons with Tukey's test (A,B) or by one-way ANOVA analysis corrected for multiple comparisons with Tukey's test (D,E). Statistical analysis for (C) was performed by a ratio paired *t-*test comparing the predicted affected fraction *fa*_*HS*,*P*_ to the observed affected fraction *fa*_*HS*,*O*_. *P<0.05; **P<0.01; ****P<0.0001; ns, not significant.

We wanted to determine if harmine also enhanced other LRAs independent of the PKC pathway. We observed that harmine also boosts the efficacy of SAHA (vorinostat), an HDAC inhibitor, to enhance viral reactivation. Harmine only boosts the MFI of SAHA-reactivated cells and not the frequency of reactivated cells (Fig. S3) because SAHA reactivation is independent of NFκB or NFAT pathways. We next tested the combinatorial effects of ingenol A, SAHA, and harmine on HIV reactivation. SAHA and harmine both boosted the percentage of ingenol A-reactivated cells and that the combination of SAHA and harmine had a more potent effect on ingenol A-induced reactivation (Fig. 6A). To determine whether this effect was synergistic, we used the Bliss Independence Model of drug synergy and found that the observed combined effects of SAHA and harmine were significantly greater than those predicted by the model indicating that the combination of SAHA and harmine is synergistic (Fig. 3C). SAHA did not boost the MFI of ingenol A-reactivated GFP^+^ cells. However, harmine did boost the MFI of ingenol A-reactivated GFP^+^ cells and the combination of harmine and SAHA had an even greater boosting effect (Fig. 6B). Similarly, we found that while harmine+ingenol A increases *gag* mRNA (Fig. 6D) and gag protein (Fig. 6E) expression compared to ingenol A alone that harmine+SAHA+ingenol A induced significantly more *gag* expression.

## DISCUSSION

**Fig. 7.**
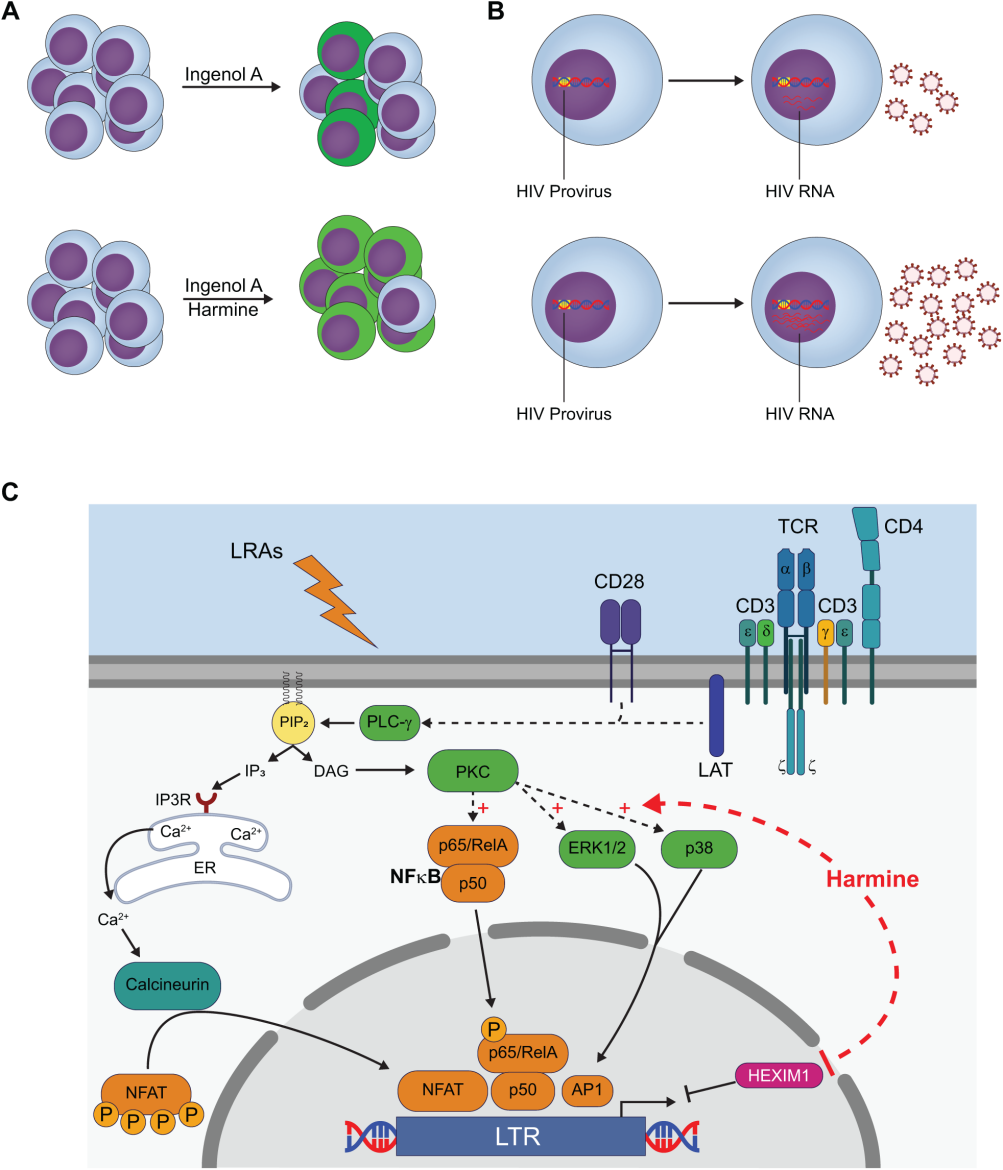
**Harmine treatment results in increased HIV reactivation by PKC-agonists.** (A) Combinatorial treatment of ingenol A and harmine results in increased frequency of GFP^+^ cells and increased MFI in GFP^+^ cells in J-Lat model. (B) Combinatorial treatment of ingenol A and harmine results in increased LTR activity and expression of HIV RNA. (C) Harmine treatment in combination with PKC agonists results in increased availability of transcription factor NFκB, increased MAPK p38 and ERK1/2 activity, and decreased HEXIM1 expression.

The primary goal of the ‘shock and kill’ strategy is to reactivate transcription of HIV so that reservoir cells will be killed by the cytopathic effects of the virus or expression of viral proteins will enable recognition and killing by the immune system. LRAs targeting a diverse range of pathways have been proposed for use but most fall short of reactivating all replication-competent cells. For the shock and kill approach to be effective all replication-competent cells must be reactivated to prevent viral rebound after removal of ART. One strategy being evaluated for improving the shock and kill method is using combinations of drugs that act synergistically to reactivate a greater number of latently infected cells (Laird et al., 2015). In the current study, we demonstrate that the plant-derived compound harmine used in combination with ingenol A increases the efficacy of latent HIV-1 reactivation. Our data show that harmine has two effects: it increases the number of cells that are reactivated by ingenol A, and it increases LTR promoter activity in reactivated cells resulting in increased viral transcripts and viral proteins (Fig. 7A,B).

In the J-Lat model, GFP acts as a surrogate measure for LTR activity. Our data demonstrate that harmine treatment results in increased GFP expression on a per-cell basis suggesting that viral gene expression would also occur. Indeed, *gag* expression was increased at both the transcript level and protein level as demonstrated by RT-qPCR and western blot, respectively (Fig. 1). Increased expression of viral proteins will make the ‘kill’ phase of the ‘shock and kill’ approach more effective. Our data also demonstrate that the combination of harmine and SAHA has a synergistic effect in boosting HIV reactivation by ingenol A (Fig. 6). The combination of these three drugs promoted reactivation of a greater number of cells and increased viral protein production in the reactivated cells (Fig. 6D,E). Interestingly, we found that harmine also boosted the reactivation effects of SAHA alone (Fig. S3), which is likely due to harmine and SAHA working on different pathways.

Previous studies have demonstrated that PKC agonists such as prostratin, bryostatin, and ingenol A work synergistically with HDAC inhibitors as well as the BRD4 inhibitor JQ1 (Brogdon et al., 2016; Díaz et al., 2015; Jiang et al., 2015; Laird et al., 2015; Martínez-Bonet et al., 2015; Perez et al., 2010; Reuse et al., 2009; Williams et al., 2004). This synergism is due to two different mechanisms for latency being targeted: availability of transcription factors and either epigenetic modifications or availability of pTEFb complexes. In our current study, we wanted to investigate whether the addition of the compound harmine would also act synergistically with PKC agonists to reactivate latently infected cells. We predicted harmine would boost reactivation by increasing availability of the transcription factor NFAT, since harmine has been shown to inhibit DYRK1A (Bain et al., 2007; Göckler et al., 2009), a negative regulator of NFAT signaling (Adayev et al., 2011; Bain et al., 2007; Egusa et al., 2011; Göckler et al., 2009). However, in the current study, we demonstrate that the boosting effect is independent of NFAT (Fig. 2).

Since harmine is a bona fide DYRK1A inhibitor and has been shown to bind to DYRK1A in crystal structures, we hypothesized that harmine may be modifying the function of DYRK1A or directing it into a different pathway. We tested if harmine treatment had any effects on signaling through the MEK/ERK and p38 MAPK pathways when used in combination with a PKC agonist. We found that harmine treatment resulted in increased signaling through these pathways in contrast to INDY. While both harmine and INDY boosted the frequency of reactivated cells after treatment with PKC agonists, only harmine boosted the MFI of the reactivated cells. This suggested that harmine may have a secondary effect on DYRK1A. However, when we knocked out DYRK1A using CRISPR/Cas9, the boosting effect of harmine was still observed (Fig. 4). This indicates that enhanced HIV reactivation seen with harmine treatment is not dependent on interaction with DYRK1A.

Microarray data demonstrated that harmine treatment reduced *HEXIM1* levels. This was confirmed at the protein level by western blot analysis (Fig. 5). HEXIM1 is a component of the pTEFb complex. The pTEFb complex consists of multiple proteins including CycT1, CDK9, and HEXIM1, and there are heterogeneous combinations of these proteins that determine the specific activity (or inactivity) of pTEFb. A 7SK snRNP complex containing HEXIM1, CDK9, CycT1, MePCE, and LARP7 along with a 7SK snRNA holds CDK9 in an inactive complex (Michels et al., 2004; Nguyen et al., 2001; Yang et al., 2001; Yik et al., 2003). A key role for HIV Tat protein is to release CDK9/CycT1 pTEFb from 7SK snRNA so that it may drive HIV mRNA transcription (Fujinaga et al., 2004; Ivanov et al., 2000; Ping and Rana, 2001). Alternatively, CDK9/CycT1 can be bound by BRD4 and this drives expression of host genes such as c-Myc (Dey et al., 2009; Jang et al., 2005; Yang et al., 2005, 2008). However, BRD4 competes with Tat for pTEFb (Li et al., 2013). It has been shown that the BRD4 inhibitor JQ1 enhances Tat-induced HIV transcription and reversal of latency (Banerjee et al., 2012; Bartholomeeusen et al., 2012). Thus, the pTEFb transcription complex acts as a three-position switch relative to HIV and host gene mRNA expression. HEXIM1-containing complexes are in the off position for all pTEFb-dependent genes. BRD4-containing complexes are in the off position for HIV transcription but the on position for host genes such as c-Myc. Finally, Tat-containing complexes are in the on position for HIV transcription.

Our microarray analysis revealed increased expression of *CCNT2*, the gene that codes for CyclinT2, and decreased expression of *HEXIM1*. While CyclinT1 is the kinase normally associated with the pTEFb complex CyclinT2 has also been reported to associate with CDK9 in the PTEF-b complex. Unlike CyclinT1, which promotes Tat activity, CyclinT2 has been reported to be inhibitory for HIV-1 transcription (Napolitano et al., 1999). It is unclear whether increased expression of CyclinT2 in our J-Lat model is having a negative effect on HIV transcription. HEXIM1, when part of the pTEFb complex, is also inhibitory for HIV-1 replication (Barboric et al., 2007; Michels et al., 2004; Yik et al., 2003). Therefore, decreased HEXIM1 expression could account for at least some of the boosting effect of harmine.

Signaling through the TCR activates NFκB and NFAT and it has been previously reported that TCR signaling enhances transcriptional elongation of latent HIV-1 by activating pTEFb through an ERK-dependent pathway (Kim et al., 2011). We found that treatment with ingenol A leads to ERK-1/2 phosphorylation and that this effect was enhanced by treatment with harmine (Fig. 3), which may be the mechanism by which harmine treatment enhances LTR promoter activity through pTEFb. Our data suggest that harmine is increasing transcriptional elongation through increased availability of pTEFb which may act synergistically with the BRD4 inhibitor JQ1.

A recent study by Booiman and colleagues demonstrated that the DYRK1A inhibitor, INDY, reactivated latently infected J-Lat cells without the use of a PKC agonist (Booiman et al., 2015). In our hands, however, treatment of J-Lat cells with DYRK1A inhibitors alone was not sufficient to reactivate latently infected cells as measured by GFP expression. However, INDY treatment alone did result in increased *gag* mRNA expression (Fig. 1D). The dose that we used for our study (20 µM) is much lower than the doses used by Booiman and colleagues. When higher doses of INDY were used in our studies, excessive toxicity for J-Lat cells was observed (not shown). Treatment with DYRK1A inhibitors only affected J-Lat cells that were stimulated with PKC agonists, SAHA, or TNF. The study by Booiman and colleagues used J-Lat clones 8.4 and A1, whereas we used clone 5A8, which may account for the differences (Booiman et al., 2015). We did not observe an increase of Gag expression by western blot in cells treated with INDY alone to correlate with the increased in gag mRNA levels. This is likely due to the higher sensitivity of qPCR compared to western blot.

Thus, the combination of ingenol A, SAHA, and harmine results in increased reactivation of latently infected J-Lat cells and increased expression of viral proteins. This is likely due to harmine treatment increasing the availability of NFκB, enhancing the effect of the PKC agonist, while simultaneously boosting the activation of the MEK/ERK pathway that leads to increased transcript elongation. When used in combination with SAHA, which inhibits HDACs and increases the availability of the LTR promoter, the result is a potent drug combination that leads to enhanced reactivation of latently infected cells. Our findings suggest that combination therapies that use currently existing LRAs would be enhanced by the addition of harmine. Additional studies are needed to determine the molecular target of harmine that results in the phenotype we have reported here before harmine is used *in vivo*.

## MATERIALS AND METHODS

### Reagents and resources

A list of key antibodies, inhibitors, commercial kits, and other reagents can be found in Table S2.

### Reactivation experiments

J-Lat 5A8 cells are treated with DMSO, harmine (20 µM), INDY (20 µM), or SAHA (5 µM) for 30 min followed by treatment with agonists for 18 h unless otherwise indicated in figure legend.

### Cell culture

J-Lat 5A8 cells, a kind gift from Warner Greene (University of California, San Francisco, CA, USA), have been previously described (Chan et al., 2013). Briefly, J-Lat 5A8 cells are Jurkat cells that are latently infected with a full-length provirus integrated into the *MAT2A* gene and have the *gfp* reporter gene in place of *nef*. A frameshift resulting in defective env production renders the cells non-infectious. Any stimulus that activates the LTR will result in transcription of *gfp*. J-Lat 5A8 cells and Jurkat luciferase reporter cells were cultured in RPMI 1640 with 2 mM L-glutamine (Corning) supplemented with 10% heat-inactivated FBS (Sigma-Aldrich, St. Louis, MO, USA) and 100 U/mL penicillin-streptomycin.

### Primary CD4^+^ T Cells

Buffy coats were obtained from Life South Community Blood Center (Gainesville, FL, USA) under approval by the Institutional Review Board at the University of Florida. PBMCs were processed as previously described (Taylor et al., 2018) and CD4^+^ T cells were enriched with the CD4^+^ T Cell Isolation Kit (Miltenyi Biotec) according to the manufacturer's instructions. CD4^+^ T cells were cultured in Advanced RPMI 1640 (Corning) supplemented with 2 mM GlutaMAX (Gibco), 10% heat-inactivated FBS (Sigma-Aldrich), and 100 U/ml penicillin-streptomycin.

### Flow cytometry and flow sorting

Cells were fixed in 2% paraformaldehyde and GFP fluorescence was measured using the BD Accuri™ C6 flow cytometer (BD Biosciences). Flow sorting was performed on unfixed cells the FACS Aria III (BD Biosciences) at the University of Florida Center for Immunology and Transplantation.

### AlamarBlue assay

Jurkat T cells were treated with different doses of harmine, INDY, or an equivalent volume of DMSO overnight. Toxicity of the inhibitors was measured by adding 10 µl of AlamarBlue^®^ Cell Viability Assay Reagent (Thermo Scientific) to 90 µl of cells and waiting for color development. Absorbance was measured at 570 and 600 nm. The percent reduction was calculated according to the manufacturer's instructions.

### Western blot

Western blots were performed as previously described (Taylor et al., 2018).

### RT-qPCR

Total RNA was isolated with the RNeasy Plus Mini Kit (Qiagen). cDNA synthesis was carried out with High-Capacity cDNA Reverse Transcription Kit (Applied Biosystems, Foster City, CA, USA) according to the manufacturer's instructions. RT-qPCR reactions were carried out in SYBR™ Select Master Mix (Thermo Fisher Scientific, Waltham, MA, USA) according to the manufacturer's instructions. RT-qPCR reactions were performed on the StepOnePlus™ Real-Time PCR System (Applied Biosystems). Gag primers used were forward: 5′- GAGCTAGAACGATTCGCAGTTA-3′; reverse: 5′- CTGTCTGAAGGGATGGTTGTAG-3′.

### Luciferase assays

Reporter cell lines were created by transducing Jurkat (E6.1) T cells with Cignal Lenti NFAT reporter (Catalogue number CLS-015 L), Cignal Lenti NFκB reporter (Catalogue number CLS-013L), or Cignal Lenti Negative Control (CLS-NCL) lentiviral particles purchased from Qiagen. An equal volume of Bright-Glo™ Luciferase Assay System (Promega) was added to an equal volume of cells. After 5 min, the cells were lysed, and the lysate was transferred to a black Costar EIA/RIA polystyrene half area 96-well plate (Corning). Luminescence was measured with the VICTOR™ X4 Multi-Plate Reader (PerkinElmer).

### Microarrays

J-Lat 5A8 cells were treated with DMSO or harmine (20 µM) for 30 min. After pretreatment, the cells were treated with either medium or PMA (10 nM) for 2 h. Total RNA was collected with the RNeasy Plus Mini Kit (Qiagen). Gene expression was assessed with GeneChip™ Human Transcriptome Array 2.0 arrays (Affymetrix) by the Interdisciplinary Center for Biotechnology Research at the University of Florida. The analysis was performed with Partek Genomics Suite v. 6.6 (Partek Inc., St. Louis, MO, USA). CEL files were imported and the raw data were subjected to multi-array average (RMA) background correction and quantile normalization. Probesets were summarized by the median polish method and the summarized signals were transformed to log base 2. A one-way ANOVA with contrast was performed to determine fold changes between PMA and PMA+harmine-treated groups. Transcripts that were significantly (step-up FDR <0.05) upregulated or downregulated by at least twofold are listed in Table S1 (coding and noncoding) and Table 1 (coding).

### Calcium flux assays

J-Lat 5A8 cells were incubated for 30 min with the Calcium Sensor Dye eFluor 514 (eBioscience) at a concentration of 2 µM with DMSO, harmine (20 µM), or INDY (20 µM). The cells were washed and resuspended in sample buffer (1X PBS, 0.1% BSA, 2 mM EDTA) containing DMSO, harmine (20 µM), or INDY (20 µM). Flow cytometry data were collected for 1 min and then ionomycin (250 nM) or ingenol A (50 nM) was added and flow cytometry data were collected for an additional 3 min. The change in the area under the curve (ΔAUC) was calculated by dividing the area under the curve after the addition of ionomycin or ingenol A divided by the AUC before the addition of ionomycin or ingenol A.

### CRISPR/Cas9 Knockout of DYRK1A

J-Lat 5A8 cells were co-transfected with two plasmids. One plasmid on the pCRISPR-CG01 backbone (GeneCopoeia) codes for recombinant Cas9 and a sgRNA (5′-GCCAAACATAAGTGACCAAC-3′) that targets exon 2 of DYRK1A. The second plasmid on the pDONOR-D01 backbone (GeneCopoeia) has a mCherry-T2A-Puro reporter cassette flanked by homology regions adjacent to the sgRNA target site in the genome. After co-transfection, the J-Lat cells were selected with puromycin (1 µg/ml).

### Drug synergy calculations

Synergy between harmine and SAHA was calculated using the Bliss Independence Model as previously described (Laird et al., 2015). The predicted fraction *fa*_*HS*,*P*_ can be calculated using the equation *fa*_*HS*,*P*_=*fa*_*H*_+*fa*_*S*_−(*fa*_*H*_ *fa*_*S*_) *fa*_*H*_=fraction of GFP^+^ cells reactivated by harmine with ingenol A *fa*_*S*_=fraction of GFP^+^ cells reactivated by SAHA with ingenol A *fa*_*HS*,*O*_=fraction of GFP^+^ cells reactivated by harmine, SAHA, and ingenol A. To determine if the combination of harmine and SAHA is synergistic, the experimentally observed fraction of cells reactivated by harmine and SAHA (*fa*_*HS*,*O*_) can be compared to the predicted fraction of reactivated GFP^+^ cells *fa*_*HS*,*P*_ using the following equation:
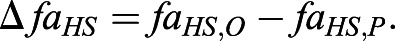


If Δ*Fa*_*HS*_ is greater than 1 then the combination is synergistic. If Δ*Fa*_*HS*_ is less than 1 then the interaction is antagonistic. If Δ*fa*_*HS*_ is equal to 0 than the mechanisms are independent of each other.

### Statistical analysis

All statistical analyses were performed using GraphPad Prism for Windows (GraphPad Software, La Jolla, CA, USA). A *P*-value of less than 0.05 was considered statistically significant.

## Supplementary Material

Supplementary information
