# Supplementary material for: Harmine enhances the activity of the HIV-1 latency-reversing agents ingenol A and SAHA
Source: Biol Open. 2020 Dec 22;9(12):bio052969. doi: 10.1242/bio.052969 (PMC7774897; doi:10.1242/bio.052969)
Supplement: Supplementary information [file biolopen-9-052969-s1.pdf]

Supplemental Figure 1

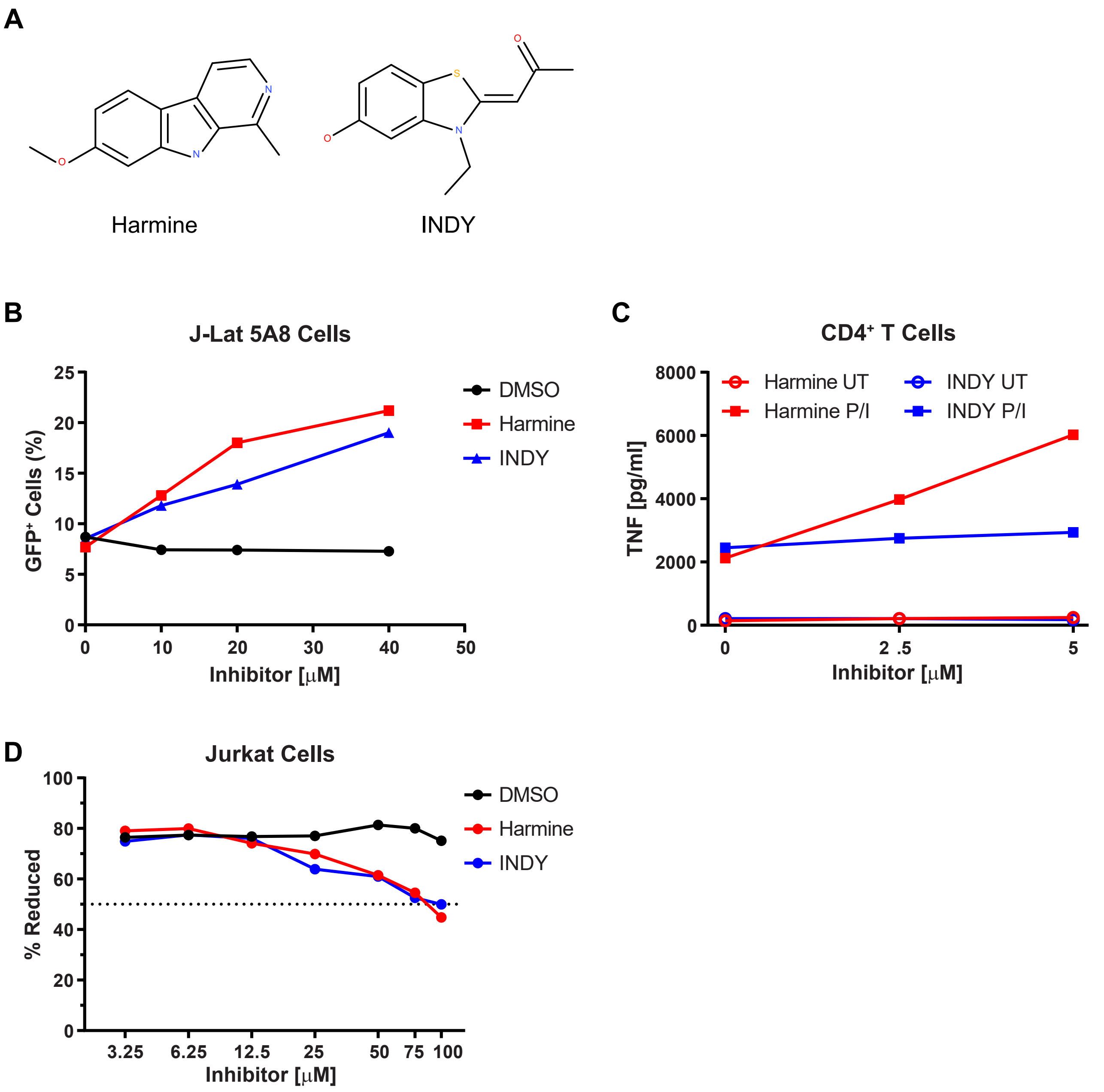

Figure S1. Dose titration of DYRK1A inhibitors.

(A) Chemical structures for harmine and INDY were generated with BIOVIA Draw v18.1 (Dassault Systèmes). **(B)** J-Lat 5A8 cells were pretreated with harmine or INDY for 30 minutes followed by activation with PMA (1.25 nM) for 18 hours (n = 1). **(C)** CD4$^+$ T cells were pretreated with harmine or INDY followed by stimulation with 1X Cell Stimulation Cocktail (81 nM PMA; 1.34 µM ionomycin) overnight. Supernatants were collected and TNF was measured by ELISA (n = 1). **(D)** Viability was measured by AlamarBlue Assay. Decreased reduction capacity indicates cellular toxicity (n = 1).

Biology Open • Supplementary information

Biology Open (2020): doi:10.1242/bio.052969 Supplementary information

Supplemental Figure 2

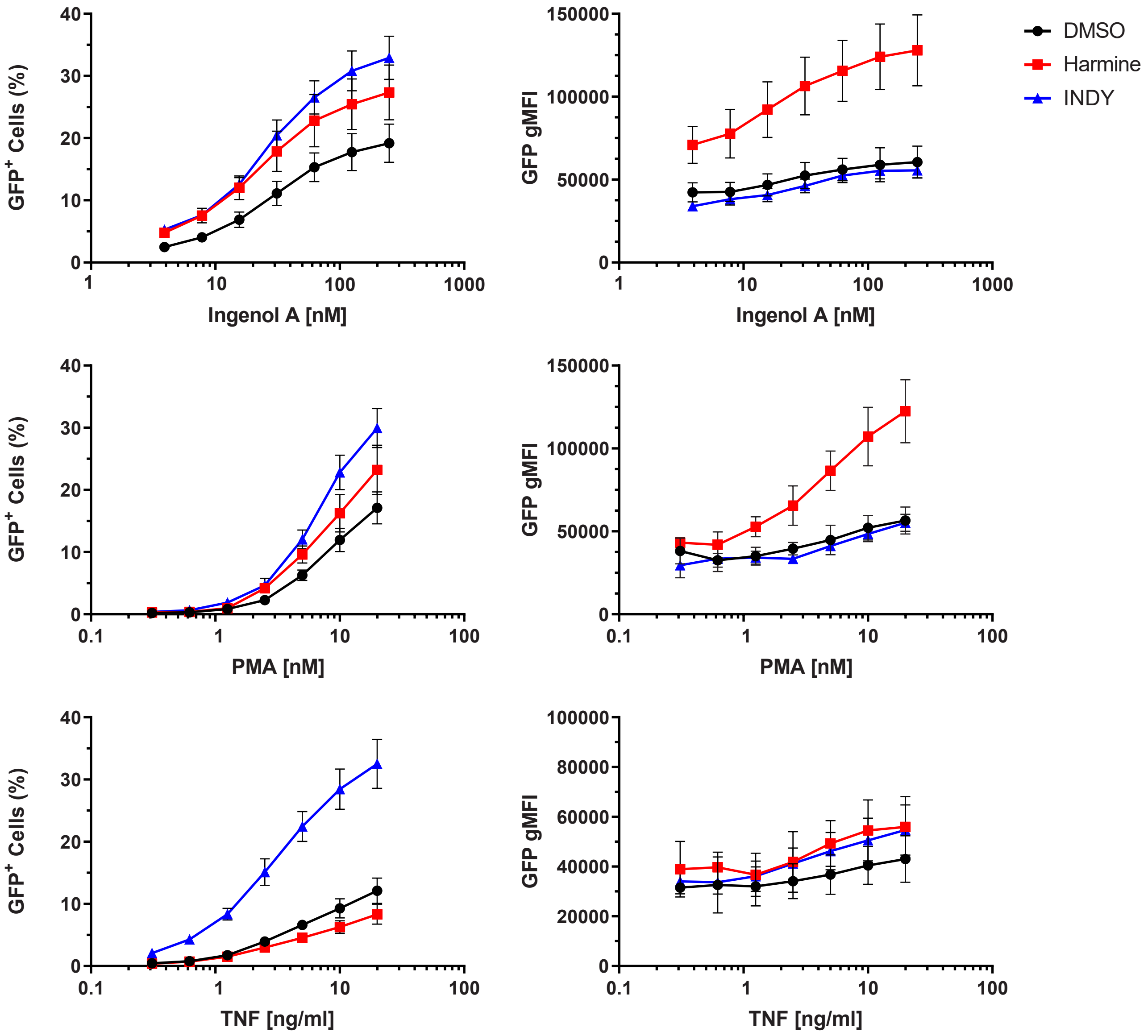

Figure S2. DYRK1A inhibitors enhance the efficacy of other PKC agonists.

J-Lat 5A8 cells were reactivated by titrating ingenol A, PMA, or TNF in the presence of inhibitors. The percentage of GFP^+ cells and the geometric mean fluorescence intensity were measured by flow cytometry (n = 3).

Biology Open • Supplementary information

Supplemental Figure 3

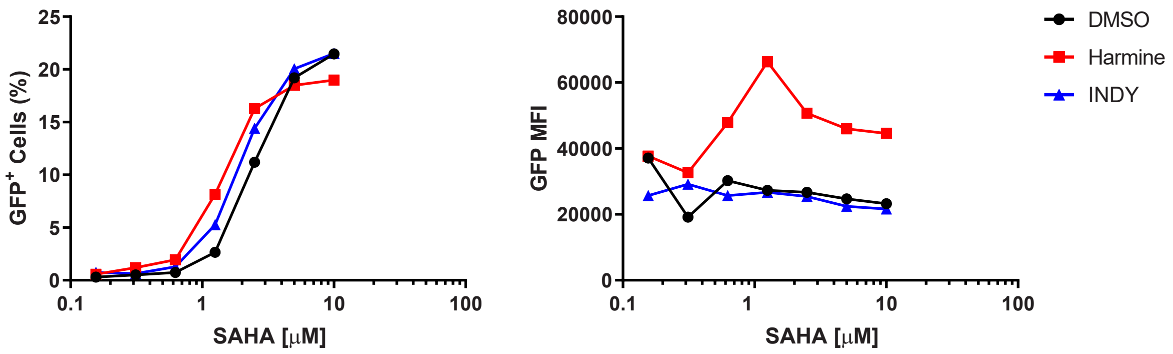

Figure S3. SAHA has no effect on the MFI of GFP+ cells but does boost the percentage of reactivated cells.

SAHA was titrated on J-Lat 5A8 cells pretreated with DMSO, harmine, or INDY for 30 minutes. The percentage of GFP+ cells and the mean fluorescence intensity of GFP+ cells was measured by flow cytometry (n = 1).

Biology Open • Supplementary information

Table S1. Transcripts significantly upregulated with PMA + harmine vs PMA treatment

Transcript ID	Gene Symbol	RefSeq	p-value	Fold-Change
TC17000580.hg.1	HEXIM1	NM_006460	1.50E-08	-3.29431
TC01005596.hg.1		---	0.00012626	-2.73307
TC15000300.hg.1	CHAC1	NM_001142776	8.53E-08	-2.52968
TC01005769.hg.1		---	0.000141118	-2.49202
TC0X000277.hg.1	USP27X	NM_001145073[a]	1.36E-05	-2.44667
TC03002656.hg.1	SKIL	BC041913	0.00013883	-2.4415
TC02004776.hg.1		---	0.000241331	-2.43804
TC02003463.hg.1		---	3.56E-05	-2.43533
TC21000594.hg.1		---	5.22E-05	-2.42017
TC01000837.hg.1	LRRC8B	NM_001134476	2.42E-07	-2.3876
TC04001570.hg.1	SLC7A11	NM_014331	1.02E-05	-2.37159
47423604_st		---	0.000545199	-2.3214
TC03002567.hg.1	RASA2	AK025305	0.000178273	-2.3009
TC09002259.hg.1		---	0.000329717	-2.28592
TC14001777.hg.1		---	4.12E-05	-2.23246
TC01005963.hg.1		---	1.56E-05	-2.23177
TC19000250.hg.1	IL27RA	NM_004843	2.71E-07	-2.1761
TC01005597.hg.1		---	2.22E-07	-2.14946
TC0X001705.hg.1	USP27X	AY672104[a]	1.46E-05	-2.13768
TC04001530.hg.1	ANKRD50	NM_001167882	3.69E-07	-2.12436
TC05003082.hg.1		---	7.27E-05	-2.11168
TC01003710.hg.1		---	0.000130796	-2.06941
TC06001830.hg.1	RAB23	NM_016277	4.59E-05	-2.03377
TC12002394.hg.1		---	0.000456661	-2.00521
TC09001204.hg.1	TMEM2	NM_001135820	1.88E-07	-2.0027
TC05001987.hg.1	SOX30	NM_007017	1.37E-07	2.10846
TC02003598.hg.1	CCNT2	NR_037649	2.86E-06	2.2142
TC02003784.hg.1		---	1.06E-05	2.23854
TC17002650.hg.1		---	3.99E-06	2.26835
TC17002649.hg.1		---	4.80E-05	2.35203
JUC_ASC002193			7.03E-05	2.35559
TC14001996.hg.1		---	7.08E-05	2.61315
TC03002295.hg.1		---	3.16E-07	2.9908
TC07001959.hg.1		---	2.99E-07	3.01515
TC04000750.hg.1		---	7.63E-07	3.75633

[a] Probes bind to the same transcript. Only NM_001145073 is included in heat map in figure 5.

Table S2. Key reagents and resources

REAGENT or RESOURCE	SOURCE	IDENTIFIER
**Experimental Models: Cell Lines**		
J-Lat 5A8 Cells	Warner Greene, University of California San Francisco	N/A
Jurkat (E6.1) Cells	ATCC	Cat# TIB-152; RRID:CVCL_0367
**Antibodies**		
Anti-HIV-1 p24	Abcam	Cat# ab9071, RRID:AB_306981
Anti-HSP90	Cell Signaling	Cat# 4877, RRID:AB_2233307
Anti-phospho-p44/42 MAPK	Cell Signaling	Cat# 5726, RRID:AB_2797617
Anti-phospho-AKT	Cell Signaling	Cat# 2965, RRID:AB_2255933
Anti-DYRK1A	Cell Signaling	Cat# 8765, RRID:AB_2797660
Anti-phospho-p38 MAPK	Cell Signaling	Cat# 4511, RRID:AB_2139682
Anti-HEXIM1	Cell Signaling	Cat# 12604, RRID:AB_2797969
Anti-β-actin	Cell Signaling	Cat# 4967, RRID:AB_330288
**Lentivirus**		
Cignal Lenti Negative Control (luc)	Qiagen	Cat# CLS-NCL
Cignal Lenti NFκB Reporter (luc)	Qiagen	Cat# CLS-013L
Cignal Lenti NFAT Reporter (luc)	Qiagen	Cat# CLS-015L
**Inhibitors**		
U0126	Cell Signaling	Cat# 9903
Harmine	Tocris	Cat# 5075
INDY	Tocris	Cat# 4997
IKK 16	MedChem Express	Cat# HY-13687
**Latency Reactivating Agents**		
SAHA (Vorinostat)	NIH AIDS Reagents Program	Cat# 12130
Ionomycin	Cell Signaling	Cat# 9995
Ingenol A (PEP005)	Tocris	Cat# 4054
PMA	Sigma	Cat# P1585
Recombinant Human TNF	PeproTech	Cat# 300-01A
**Commercial Kits**		
RNeasy Plus Mini Kit	Qiagen	Cat# 74136
High Capacity cDNA Reverse Transcription Kit	Applied Biosystems	Cat# 4368814
SYBR Select Master Mix	Thermo Fisher	Cat# 4472918
Calcium Sensor Dye eFluor 514	eBioscience	Cat# 65-0859

Biology Open • Supplementary information

Bright-Glo Luciferase Assay System	Promega	Cat# E2620
BD OptEIA Human TNF ELISA Set	BD Biosciences	Cat# 555212
AlamarBlue Cell Viability Assay Reagent	Thermo Fisher	Cat# 88951
Cell Stimulation Cocktail (500X)	eBioscience	Cat# 00-4970
$CD4^+$ T Cell Isolation Kit, Human	Miltenyi Biotec	Cat# 130-096-533
**Deposited Data**		
Microarray Data	This Paper	GEO# GSE136172

Biology Open • Supplementary information
